# Research on smoke simulation with vortex shedding

**DOI:** 10.1371/journal.pone.0269114

**Published:** 2022-06-16

**Authors:** Rui Tao, Hongxiang Ren, Delong Wang, Xiangen Bai

**Affiliations:** 1 Navigation College, Dalian Maritime University, Dalian, China; 2 Merchant Marine College, Shanghai Maritime University, Shanghai, China; University of New South Wales, AUSTRALIA

## Abstract

The Lagrangian vortex method has the advantage of producing highly detailed simulations of fluids such as turbulent smoke. However, this method has two problems: the construction of the velocity field from the vorticity field is inefficient, and handling the boundary condition is difficult. We present a pure Lagrangian vortex method, including a nested grid to accelerate the construction of the velocity field, and a novel boundary treatment method for the vorticity field. Based on a tree structure, the nested grid algorithm considerably improves the efficiency of the velocity computation while producing visual results that are comparable with the original flow. Based on the vortex-generating method, the least square method is used to compute the vorticity strength of the new vortex elements. Further, we consider the mutual influence between the generated vortex particles. We demonstrate our method’s benefits by using a vortex ring and various examples of interaction between the smoke and obstacles.

## Introduction

Incompressible flows can be widely observed in our daily life, such as smoke from burning wood and the vortices generated by water flowing through obstacles. As a main research direction in fluid simulation, smoke simulation has drawn substantial attention in industry and academia. The effective use of limited computing resources to obtain a more realistic effect of smoke movement is still challenging.

Various categories of numerical methods were developed to explore different types of complex and changing smoke phenomena. In terms of computing variables, these methods are classified for solving velocity, vorticity, wave function, and so on. In terms of discretization forms, these methods are classified as Lagrangian methods, Eulerian methods, and Lagrangian–Eulerian hybrid methods. Because of their unique advantages, these methods can be used to numerically simulate various flows.

The Lagrangian vortex method has a unique advantage in the simulation of unsteady flows where the vortex structures play a leading role, such as the dynamical evolution of a forced shear layer [1], an impulsively started rigid body [2], and certain interacting coherent vortical structures [3, 4]. First, this method exhibits all the merits of the Lagrangian method. With the material elements themselves being discrete elements, the Lagrangian vortex method does not have to construct complicated Eulerian meshes for flows with complex geometries. Moreover, it significantly decreases numerical dissipations [5] by avoiding interpolating between grids with the physical quantities stored on material elements. Second, the Lagrangian vortex elements can be placed adaptively during the process of discretizing the vorticity field, with the number of vortex elements placed being proportional to the vorticity strength. In addition, the continuous incompressible velocity field can be built by using the Biot–Saviot(BS) law from the discretized vorticity field. Thus, a continuous solenoidal velocity field that fills the entire computational domain can be reconstructed with relatively fewer discretized vortex elements needed for dynamical evolution [6].

However, unlike the velocity-based Lagrangian method [7, 8], the Lagrangian vortex method has not yet been applied widely in industry and academia for the following reasons. First, the interaction between vortex elements is non-local, leading to higher complexities of high-precision and high-speed algorithms for large-scale simulations [9]. Second, the boundary condition of the flow field is generally given based on the velocity field, making the conversion of the velocity to vorticity boundary conditions necessary [6, 10]. This conversion could be nontrivial, particularly for moving boundaries.

To solve the first of the aforementioned problem, we use a nested grid to group the vortex particles based on their positions and treat the vortex particles in the same group as one super vortex particle in an approximate manner. When calculating the velocity at a given position, we traverse the super vortex particles instead of all the original vortex particles. Thus, we can reduce the number of traversals to decrease the computational cost. We use the vortex–generating method to handle the boundary condition for the second problem. Unlike the momentum form of the Navier–Stokes equation, the vorticity at the boundary cannot be directly set. The plane method [5, 11] in classic airfoil theory is only suitable for simple non-slip boundary conditions. In this study, the generated vortex particles are placed on the boundary to satisfy the flow’s non-through and non-slip boundary conditions. The vorticity of the generated vortex particles is implicitly determined by solving a linear system. Our contributions are summarized as follows:

1. A parallel nested grid method that groups the vortex particles to decrease the number of vortex particles traversed during the velocity computation, thus reducing the computational cost;
2. A novel boundary treatment method based on optimization. The least-square method is used to compute the vorticity strength of the generated vortex particles, satisfying the non-through and non-slip boundary conditions;
3. A pure Lagrangian vortex dynamics framework that simulates complex vortical smoke phenomena with moving obstacles.

## Related work

Modeling the dynamic evolution of smoke is a laborious task. Owing to the development of algorithms and hardware, researchers have adopted physics-based methods to simulate smoke. Stable Fluids [12] is a classic method based on the Eulerian method. Although this method produces massive numerical dissipation, it can obtain unconditional numerical stability with large time steps. Subsequently, numerous methods were proposed to reduce the numerical dissipation, such as the BFECC [13], MacCormack [14], advection–reflection [15], “BiMocq2” [16], and vorticity confinement [17–19] approaches. A common feature of the Eulerian method is that the fluid domain should be discretized in advance. Hence, the Eulerian method is not suitable for smoke simulation in open spaces.

Among the Lagrangian methods, the most widely studied methods are the smooth particle hydrodynamics (SPH) and Lagrangian vortex methods. The advantage of the SPH method is its high computational efficiency. This is because the SPH method does not have to solve the global Euler equation. However, its accuracy relies on the massive distribution of particles, and this is not suitable for smoke simulation in large spaces. To solve this problem, Ren et al. [20] further improved the performance of SPH smoke simulation by solving for the visible smoke without involving the transparent air.

The Lagrangian vortex method is the most efficient method for simulating complex flows with sparse date and minimal numerical dissipation. This method discretizes the vorticity field on the Lagrangian vortex element and implements a velocity field by using the BS law without the numerical dissipation that is associated with projection. The flow data can be stored on vortex particles [9, 21, 22], vortex filaments [3, 23–26], and vortex surfaces [27–29]. Based on the connectivity of the mesh, the vortex surfaces can be used to track the fluid surfaces. However, this discrete form cannot deal with the topological changes of vortex structures. The vortex filament discretizes the vorticity field on the closed curves, which naturally represents the vortex structure. A fundamental problem with the vortex filament method is the complicated curve repair operation to the topological changes in the filament, making large-scale parallel computing challenging. The vortex particle method, also known as the vortex blob method, generally replaces point vortices with certain vortex cores to remove the singularity in the kernel function. The vortex core has various shapes, such as sphere, ellipsoid, or small vortex sheet. Compared with the vortex filament and vortex surfaces, it has a lower computational overhead and better stability but with decreased accuracy.

One common problem of the Lagrangian vortex method is that the integration operation’s time complexity, from the vorticity field to the velocity field, is *O*(*N*^2^). To overcome this problem, some classic solutions were proposed, such as the Fast Multipole Method (FMM) [30], Particle-Particle Particle-Mesh (PPPM) [9], and Octree-Based Method (OBM) [22]. The FMM can reduce the integral operation’s time complexity to *O*(*N*) and guarantee sufficient accuracy. However, this algorithm is considerably complex and difficult to deploy on new hardware, making it impossible to be widely used in industries. The PPPM method has a computational time complexity of *O*(*NlogN*) at the expense of partial accuracy and is relatively easy to implement. The OBM method uses a hierarchical tree data structure to group vortex particles, and the computational time complexity is *O*(*NlogN*). However, the tree structure that carries the vortex particles’ spatial distribution information makes the parallelization of the algorithm challenging.

The boundary treatment is notoriously problematic in the Lagrangian vortex method. In computer graphics, researchers generally place vortex layers at the boundary to satisfy the non-slip and non-through boundary conditions. Based on this idea, Chorin [21] proposed a vortex-generating vortex method to deal with the boundary conditions of vortical flows. Park [31] obtained a reasonable vorticity distribution on the solid boundary by solving a system of 3*K*_*g*_ equations, where *K*_*g*_ is the number of generated vortex elements. Vines [32] reduced the size of the linear system to *K*_*g*_ by applying only a non-through boundary condition to the normal of the solid, thereby solving the boundary integral problem. Appropriate boundary conditions can reflect the physical mechanism of vorticity generation on the solid. An SPH turbulence model can be used to deal with boundary conditions of vortex filaments [33].

## Materials and methods

### Vortex dynamics

#### Vortex particle method

The Navier–Stokes equation can be written in two forms: the velocity (***u***)–pressure (*p*) form and the velocity (***u***)–vorticity (***ω*** = **∇** × ***u***) form. The former focuses on tracking the change in momentum to solve the pressure, viscous force, or other external forces in the equation. In this study, we use the latter to simulate the smoke by using the following vorticity equation:

$$\begin{array}{c} \nabla \cdot u=0, \end{array}$$ (1)

$$\begin{array}{c} \frac{D\omega }{Dt}=\left(\nabla u\right)\cdot \omega +\nu \nabla^{2}\omega , \end{array}$$ (2)

where *ν* is the viscosity coefficient. Eq (1) expresses the incompressibility of the fluid. In Eq (2), the pressure term disappears. The left side of Eq (2) shows the change rate of vorticity under the Lagrangian viewpoint, which can be further expressed as *∂* ***ω***/*∂t* + (***u*** · **∇**)***ω***. Furthermore, (***u*** · **∇**)***ω*** is the advection term. The right side of Eq (2) shows the stretching and diffusion terms, from left to right. The stretching term does not exist in 2D space. In 3D space, as a key element of turbulent motion, it can produce local intensification and reorientation of the vorticity. The diffusion term describes the fluid’s vorticity diffusion due to friction, which can smooth the diversity of fluid movement and slow down the motion of flow. The diffusion term can be solved by employing the particle strength exchange (PSE) method [34], which is not discussed in this paper.

Based on the Lagrangian viewpoint, the vorticity field is discretized on a set of vortex particles; this is consistent with the fact that the non-zero vorticity is generally concentrated in the flow trajectory [35]. The vorticity strength carried by the vortex particles can be computed by using following equation:

$$\begin{array}{c} \Gamma_{i}=\int_{V_{i}}^{}\omega dV\approx \omega_{i}V_{i}, \end{array}$$ (3)

where **Γ**_*i*_, ***ω***_*i*_, and *V*_*i*_ is the vorticity strength, vorticity, and volume of the *i*^*th*^ vortex particle, respectively.

#### Stretching

The methods used for solving the stretching term can be roughly classified into two categories. An explicit method is to discretize the velocity on the grid and compute the Jacobi matrix which is all partial derivatives of all components (*x*, *y*, *z*) of velocity ***u*** = (*u*, *v*, *w*). The accuracy of this method is considerably low because of the interpolation. Another approach is to capture the vortex structure’s stretching by calculating the deformation of the geometric structure, such as the vortex ring or vortex sheet in the flow, which cannot be directly applied in the vortex particle method.

We use the vortex segment approach to solve the stretching term. To use vortex segments, which naturally represent the vortices’ geometric structure, we build a bridge between the vortex particles and the vortex segments. Each vortex segment is a small cylinder of length *h* and constant circulation *κ*. The segment has the same direction as the vorticity strength and two endpoints, ***x***_*l*_ and ***x***_*r*_. A vortex particle of vorticity strength **Γ**_*i*_ can be translated to a vortex segment of unit direction $T_{i}=\frac{\Gamma_{i}}{\left\|\Gamma_{i}\right\|}$, length *h*_*i*_, and circulation $\kappa_{i}=\frac{\left\|\Gamma_{i}\right\|}{h_{i}}$.

We first convert the vortex particles into vortex segments and then update the ends of the vortex segments by applying the following equation:

$$\{\begin{array}{l} x_{i,l}^{n+1}=x_{i,l}^{n}+\Delta tu_{i,l}^{n}\\ x_{i,r}^{n+1}=x_{i,r}^{n}+\Delta tu_{i,r}^{n}, \end{array}$$ (4)

where ***u*** is the ends’ velocity that can be calculated by using Eq (8).

Finally, we translate the vortex segments back into vortex particles with updated vorticity strengths. If we directly set the vortex particle with vorticity strength ${\tilde{\Gamma_{i}}}^{n+1}=\kappa_{i}\left(x_{i,r}^{n+1}-x_{i,l}^{n+1}\right)$, even if the time step is small, the simulation diverges quickly. The reason is that, although the magnitude of velocity is small, the gradient of the velocity field can be large in a turbulent flow. This fact makes the velocity difference between the two ends significantly large, resulting in an unstable simulation. Therefore, we weight ${\tilde{\Gamma_{i}}}^{n+1}$ and $\Gamma_{i}^{n+1}$ to stabilize the simulation and obtain the final vorticity strength:

$$\begin{array}{c} \Gamma_{i}^{n+1}=\left(1-\delta \right)\Gamma_{i}^{n}+\delta {\tilde{\Gamma_{i}}}^{n+1}, \end{array}$$ (5)

where *δ* ∈ (0, 1) is the interpolation coefficient. Notice that in our vortex segment approach, we smooth the update of the vorticity instead of ${\tilde{\Gamma_{i}}}^{n+1}$ to preserve sharp features as much as possible.

#### Velocity computation

In the Lagrangian vortex method, the velocity field can be obtained from the vorticity field by using the BS law:

$$\begin{array}{c} u\left(p\right)=\int_{R^{d}}^{}\omega \left(x\right)\times \frac{p-x}{{\left\|p-x\right\|}^{d}}dx, \end{array}$$ (6)

where ***p*** is the inquiring position, and *d* is the computational domain’s dimension. In this study, the integration in Eq (6) is equivalent to the summation of all vortex particles

$$\begin{array}{c} 2D:u\left(p\right)=\sum_{i=1}^{K_{v}}\frac{\left(0,0,\Gamma_{i}\right)\times \left(p-x_{i}\right)}{2\pi {\left\|p-x_{i}\right\|}^{2}}, \end{array}$$ (7)

$$\begin{array}{c} 3D:u\left(p\right)=\sum_{i=1}^{K_{v}}\frac{\Gamma_{i}\times \left(p-x_{i}\right)}{4\pi {\left\|p-x_{i}\right\|}^{3}}, \end{array}$$ (8)

where *K*_*v*_ is the number of vortex particles, and ***x***_*i*_ and **Γ**_*i*_ are the position and vorticity strength of the *i*^*th*^ vortex particle, respectively. Moreover, Γ_*i*_ is a scalar in 2D. The BS law has the following property: the formula’s divergence-free velocity field is not unique. Consequently, the corresponding vorticity field is uniquely determined for a given velocity field. This property allows us to add a harmonic velocity field.

However, the BS law has two problems. One is the expensive numerical integration. We introduce a solution to this problem in Section. Another problem is that vortex particle self-induction can cause a singularity and numerical instability. Researchers have proposed certain methods to modify the BS law to circumvent this problem. Angelidis and Neyret replaced the BS kernel with a radial basis function [36], which is defined smoothly around the centroid. This makes it easier to analyze the integral.

Chorin [21] proposed the concept of a “vortex cluster,” which is a small area with a high concentration of vorticity. The shape of the area can be circular, elliptical, or vortex sheet. The combination of the vortex clusters and different non-singularized kernel functions achieves different smoothing effects. In this study, an artificial smoothing parameter, *σ*, is added to Eqs (8) and (9) is our modified BS formula.

$$\{\begin{array}{l} u(p)=\sum_{i=1}^{K_{v}}\frac{\Gamma_{i}\times (p-x_{i})}{K}\\ K=4\pi {\left\|p-x_{i}\right\|}^{3}+\sigma^{2}. \end{array}$$ (9)

**Fig 1:**
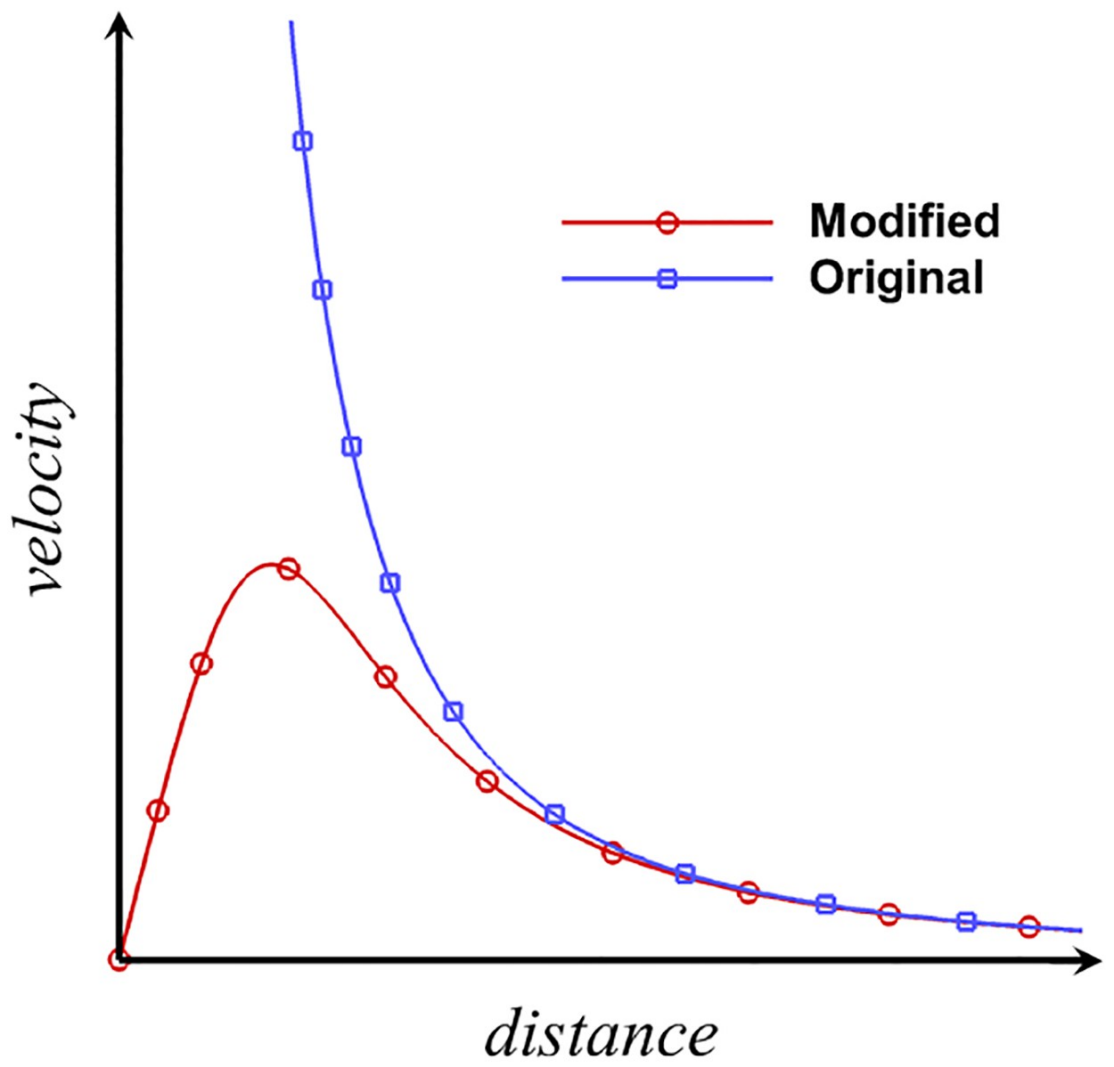
Induced velocity profile of the Biot–Savart formula. The horizontal axis is the distance between the inquiring position and the vortex particle, and the vertical axis is the magnitude of the induced velocity.

When the inquiring position, ***p***, is far away from the vortex particles, *K* ≈ 4*π* ‖***p*** − ***x***_*i*_‖^3^. When ***p*** is close to the vortex particles, the properties expressed by Eq (9) are similar to the solid vortex core. Fig 1 shows the velocity profile induced by the modified BS law.

### Fast summation

A major problem of the BS law is the time-consuming summation. As described by Eq (8), we need to traverse *K*_*v*_ vortex particles when calculating the velocity of the inquiring position. This means that the time complexity of calculating the velocity of *K*_*v*_ vortex particles is $O(K_{v}^{2})$. When a phenomenon requires massive vortex particles to show the details, the computational cost is extremely high.

By using the BS law, we derive an approximate method for reducing the traverse number in summation. According to Eq (9), the velocity is inversely proportional to the square of the distance. This property shows that the distant vortex particles have less influence on the final result, whereas the closer vortex particles have a significant contribution. If the distance between the vortex particles is much shorter than their distance to the inquiring position, the distance between these vortex particles and the inquiring position can be regarded as approximately equal. By employing the approximation, we can obtain

$$\begin{array}{c} u\left(p\right)=\frac{1}{4\pi }\sum_{i=1}^{K_{s}}\frac{\Gamma_{i}\times \left(p-x_{i}\right)}{{\left\|p-x_{i}\right\|}^{3}}\approx \frac{\left(p-\bar{x}\right)}{4\pi {\left\|p-\bar{x}\right\|}^{3}}\times \sum_{i=1}^{K_{s}}\Gamma_{i}, \end{array}$$ (10)

where *K*_*s*_ is the number of vortex particles, ***p*** is the inquiring position, and $\bar{x}$ is the weighted average position of the vortex particles.

By applying Eq (10), we find that a group of vortex particles have roughly the same contribution as a super vortex particle towards a distant inquiring position. Therefore, based on their positions, we can group the vortex particles and treat those in the same group as a super vortex particle. Thus, when calculating the velocity field, we only need to traverse the super vortex particles. This can reduce the computational overhead.

In this study, a nested grid algorithm that is similar to a tree structure is proposed to group vortex particles. The nested grid is a set of grid layers, ***G*** = {*G*_*i*_|*i* = 1, 2, …, *K*_*n*_}, with different resolutions and the same bounding box, where *K*_*n*_ is the number of grid layers. The resolution relationship between grid layers *G*^*i*^ and *G*^*i*+1^ is ${\left(K_{r}^{i+1}\right)}^{d}={\left(2*K_{r}^{i}\right)}^{d}$, where *d* is the spatial dimension. It means that each cell in grid layer *G*^*i*^ corresponds exactly to the 2^d^ cells in *G*^*i*+ 1^. The consistency in space is convenient for program implementation. All grid cells are regarded as a super vortex particle, and its vorticity and position are calculated by using Eq (11).

$$\{\begin{array}{l} x_{s}=\frac{\sum_{i=1}^{K_{s}}\left\|\Gamma_{i}\right\|x_{i}}{\sum_{i=1}^{K_{s}}\left\|\Gamma_{i}\right\|}\\ \Gamma_{s}=\sum_{i=1}^{K_{s}}\Gamma_{i}, \end{array}$$ (11)

where **Γ**_*s*_ and ***x***_*s*_ are the vorticity strength and position of the super vortex particle; and *K*_*s*_, **Γ**_*i*_, and ***x***_*i*_ are the total number, vorticity strength, and position of the *i*^*th*^ vortex particles in the cell, respectively.

Based on the spatial consistency of different grid layers, we design the traversal manner of the super vortex particles as follows:

1. Starting from the first grid layer, *G*^*i*=1^, traverse all the super vortex particles, $C_{s}^{i=1}$ in *G*^*i* = 1^, that is, the grid cells. Find all cells, $C_{s}^{\times }$, that do not contain the inquiring position, ***p***, and directly calculate their induced velocity. Then, find cell $C_{s}^{*}$ that contains the inquiring position, ***p***, and enter the next grid layer, *G*^*i*=2^.
2. Starting from the second grid layer, *G*^*i*=2^, traverse only the cell set, $C_{s}^{i}$, that corresponds to $C_{s}^{*}$ in the upper grid layer. Find the cell set, $C_{s}^{\times }$, that does not contain the inquiring position, ***p***, in $C_{s}^{i}$ and compute their induced velocities to accumulate the result with the upper grid layers. Then, find cell $C_{s}^{*}$ containing the inquiring position, ***p***, enter the next grid layer, *G*_*i*+1_, and repeat the second step until the penultimate grid layer.
3. In the last grid layer, $G^{K_{n}}$, find the cell set corresponding to grid cell $C_{s}^{*}$ in the upper grid layer and compute their induced velocity.

**Fig 2:**
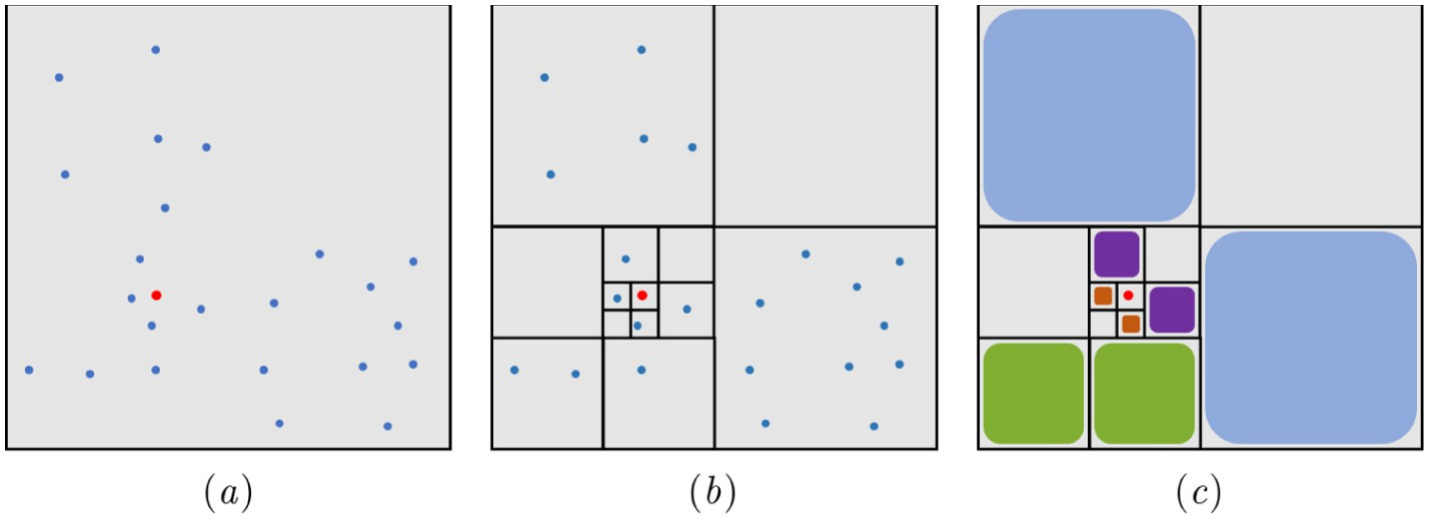
Schematic diagram of the nested grid algorithm. (a) Distribution of vortex particles; the red point is the inquiring position, *p*, and the blue points are the vortex particles. (b) Structure of the nested grid. (c) Distribution of super vortex particles.

We use a 2D example to illustrate the operation of the nested grid. First, we group the vortex particles depending on their distribution in the computation domain, as shown in Fig 2(b). The vortex particles in the same group are regarded as a super vortex particle. Fig 2(c) shows the distribution of super vortex particles in different grid layers for calculating the velocity of ***p***. Thus, the time complexity of calculating the velocity of ***p*** is reduced from *O*(*K*_*v*_) to *O*(*logK*_*v*_). For games or real-time applications, performance and effects are more important than accuracy. Although our method loses some accuracy, it substantially improves computational efficiency and can obtain satisfactory visual results.

### Boundary treatment

We use the vortex-generating method to deal with the boundary conditions for the Lagrangian vortex method. Owing to friction and viscosity, vortices are generated on the surface when the flow passes the obstacle. The vortex-generating method is consistent with this fact. The method can be divided into two steps: (1) compute the position and direction of the generated vortex particles; (2) calculate the magnitude of vorticity strength of the generated vortex particles. From simple to complex, we take the 2D static boundary example to introduce our method and then present the dynamic boundary example. Whether it is a static or dynamic boundary, we follow a principle when dealing with boundary conditions: the obstacle velocity equals the flow velocity.

**Fig 3:**
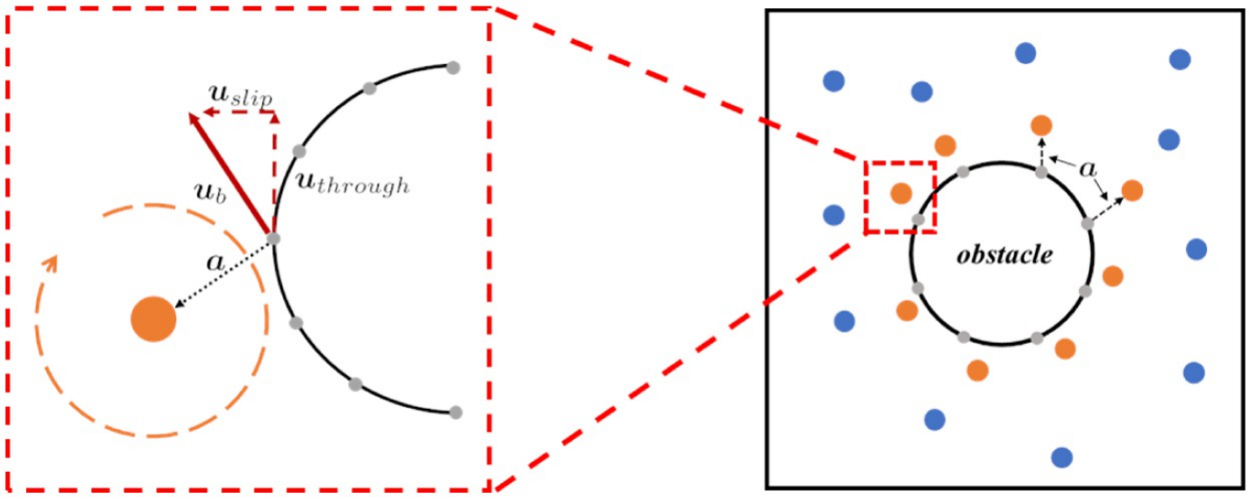
Our boundary treatment method. The gray point is the boundary sampling point; the orange point is the generated vortex particle; $v_{b}$ is the induced velocity of flow at the boundary sampling position.

First, we introduce the method of computing position ***x***_*g*_ and direction ***t***_*g*_ of the generated vortex particles. As shown in Fig 3, we sample some boundary points that are represented by the gray points with position ***x***_*b*_. The number, *K*_*g*_, of boundary sampling points is the same as the number of generated vortex particles. To offset the slip and through velocity of the flow at the boundary sampling point, ***t***_*g*_ should be tangent to the velocity, ***u***_*f*_, of the flow at the boundary and the normal, ***n***, of the solid surface. By normalizing the vorticity direction, we can obtain

$$\begin{array}{c} t_{g}=\frac{u_{f}\times n}{\left\|u_{f}\times n\right\|}. \end{array}$$ (12)

We place the generated vortex particle near the boundary along vector $a=\alpha \frac{t_{g}\times u_{f}}{\left\|t_{g}\times u_{f}\right\|}$, as shown in Fig 3. As a result, ***x***_*g*_ = ***x***_*b*_ + ***a***. The placement makes the generated vortex particle’s induced velocity at the boundary opposite to ***u***_*f*_. In our method, *α* is set artificially. The larger *α* is, the bigger the vorticity strength of the generated vortex particle, and vice versa.

Next, we use the least square method to compute the magnitude, Γ_*g*_, of the vorticity strength of the generated vortex particles. Assuming that the velocity of background flow is ***u***_∞_, we consider that the summation of the induced velocity, ***u***_*g*_, of the generated vortex particles to the boundary and ***u***_*f*_ + ***u***_∞_ is equal to the velocity, ***u***_*b*_ = 0, of the solid under ideal conditions. Therefore, the problem has been reduced to finding a minimizer of the total velocity.

$$\begin{array}{c} \Gamma_{g}={\text{argmin}}_{\Gamma_{g}}\sum_{i=1}^{K_{g}}{\left\|u_{g}^{i}\left(\Gamma_{g}\right)+u_{f}^{i}+u_{\infty }^{i}\right\|}^{2}. \end{array}$$ (13)

We derive Eq (13) as

$$\begin{array}{c} A\Gamma_{g}=BU, \end{array}$$ (14)

where ***A*** = [**BB**^*T*^]^-1^, $B=\sum_{i=1}^{K_{g}}b\left(x_{b}^{i},x_{g}\right)$, $b\left(x_{b}^{i},x_{g}\right)={\left[b_{1}\left(x_{b}^{i},x_{g}^{1}\right),\ldots b_{N}\left(x_{b}^{i},x_{g}^{K_{g}}\right)\right]}^{T}$, $U=u_{g}+u_{f}+u_{\infty }$, and $b_{j}\left(x_{b}^{i},x_{g}^{j}\right)=\left(t_{g}^{j}\Gamma_{g}^{j}\right)\times \left(x_{b}^{i}-x_{g}^{j}\right)/\left(2\pi {\left\|x_{b}^{i}-x_{g}^{j}\right\|}^{2}+\sigma^{2}\right),j=1,\ldots K_{g}$, which is the boundary velocity induced by the generated vortex particles with the unit vorticity strength vector. Eq (14) can be solved as

$$\begin{array}{c} \Gamma_{g}=A^{-1}BU, \end{array}$$ (15)

where $A={\left[\sum_{i=1}^{K_{g}}b\left(x_{b}^{i},x_{g}\right)b{\left(x_{b}^{i},x_{g}\right)}^{T}\right]}^{-1}$.

Different from the static boundary, we need to compute the velocity, ***u***_*b*_, of the solid for the dynamic boundary treatment. We also need to update the boundary sampling points in each time step, which can be computed by using Eq 16. The rigid body’s motion is divided into two parts: the center translation and the rotation relative to the center. The position of the boundary sampling points is found as

$$\begin{array}{c} x_{b}\left(t\right)=x_{c}\left(t\right)+[x_{b}\left(0\right)-x_{c}\left(0\right)]M_{c}{\left(t\right)}^{T}, \end{array}$$ (16)

where $x_{c}\left(t\right)$ and $M_{c}\left(t\right)\in R^{d\times d}$ are the center and rotation matrix of the rigid body, respectively. *d* is the spatial dimension, and $[(x_{g}\left(0\right)-x_{c}\left(0\right)]M_{c}{\left(t\right)}^{T}$ represents the rotation of the rigid body. Then, we take the time derivative of $x_{b}\left(t\right)$ to determine the velocity, $u_{b}$, of the rigid body at the boundary sampling point.

Substituting Eq (16) and $u_{b}$ into Eq (15) yields the magnitude of the vorticity strength of the generated vortex particles at the dynamic boundary:

$$\begin{array}{c} \Gamma_{g}=A^{-1}BM_{c}^{T}U^{*}, \end{array}$$ (17)

where U* = ***u***_*g*_ + ***u***_∞_ + ***u***_*f*_ − ***u***_*b*_.

### Time integration scheme

Our time integration scheme can be summarized as follows. We first update the position of the vortex and tracer particles. In our method, tracer particles are used for visualization. Then, we handle the boundary condition and solve the stretching and diffusion terms. Finally, we construct the velocity field from the vorticity field by using the modified BS law and the background flow. In each time step, we update the states of the flow by using the following steps, as depicted in S1 Fig:

1. Advection. Calculate the induced velocity by using Eq (9) and the advect vortex and tracer particles by using the fourth-order Runge–Kutta method.
2. Boundary treatment. Perform collision detection between the particles and the solid, and compute the position and vorticity strength of the generated vortex particles.
3. Stretching. Use the vortex segment method to update the vorticity strength of the vortex particles.
4. Diffusion. Use the PSE method to solve the diffusion term.
5. Velocity computation. Obtain the bounding box based on the distribution of particles. Then, calculate the velocity of the vortex and tracer particles using our nested grid algorithm.
6. Background flow. Add the velocity of background flow to the particles.

## Results

### Parameter study

**Fig 4:**
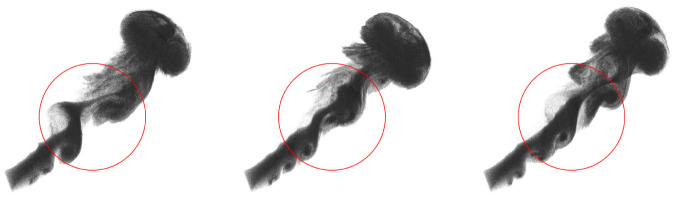
Rising smoke with three different values of *V*. Left: *V* = 64*υ*^3^; middle: *V* = 8*υ*^3^; right: *V* = *υ*^3^. Here, *υ* is 6.28*10^−3^. And the difference of the vortices are marked by the red circles.

We conduct two sets of simulations with different parameter values to better understand the impact of the vortex particle’s volume *V* and the stretching coefficient *δ* on the simulation. First, we simulate the rising smoke with three different values of *V* (as shown in Fig 4). In this example, the computational domain is approximately 20 * 20 * 20. We emit a vortex circle (consisting of a set of vortex particles) every 5-timesteps, whose normal towards the upper right. From left to right in Fig 4, *V* equal to 64*υ*^3^, 8*υ*^3^ and *υ*^3^, respectively. Here, *υ* is 6.28 * 10^−3^. To ensure the vorticity strength of the three flows are the same, the vortex particles’ number of the vortex circle is 1000, 2000 and 4000 from left to right, respectively.

We observe that the overall motion trends of the three cases are consistent, which shows that the volume of the vortex particle does not change the macroscopic behavior of the flow. In the fine scale, the difference of the vortices with different *V* are marked by the red circles. The vortices generated by the vortex particles with big *V* merge into larger vortices earlier. In contrast, the vortices in the flow with small *V* maintain longer. It is because the flow with small *V* has more vortex particles, which samples the vorticity field more uniformly. However, the increase in the number of vortex particles also brings more computational overhead. Therefore, we should balance the computational speed and animation quality according to the actual situation.

**Fig 5:**
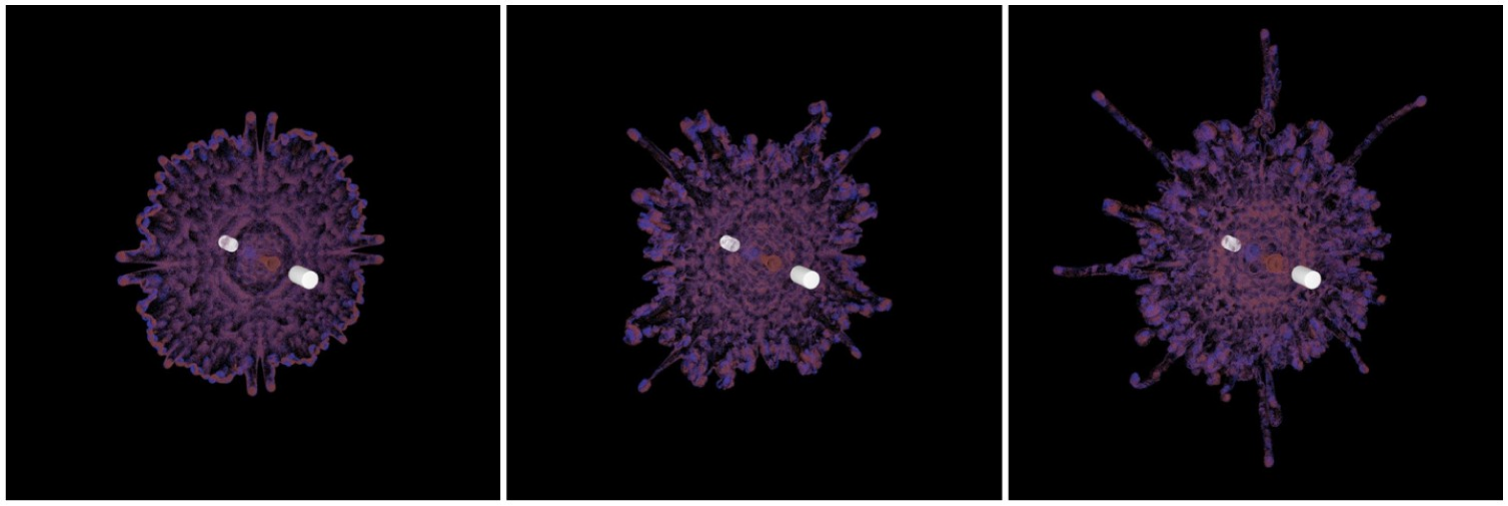
Colliding vortex rings with different *δ* at frames 400. Two regions of red and blue ink are initialized to show the motion of vortex rings. Left: *δ* = 0.4; middle: *δ* = 0.8; right: *δ* = 1.

**Fig 6:**
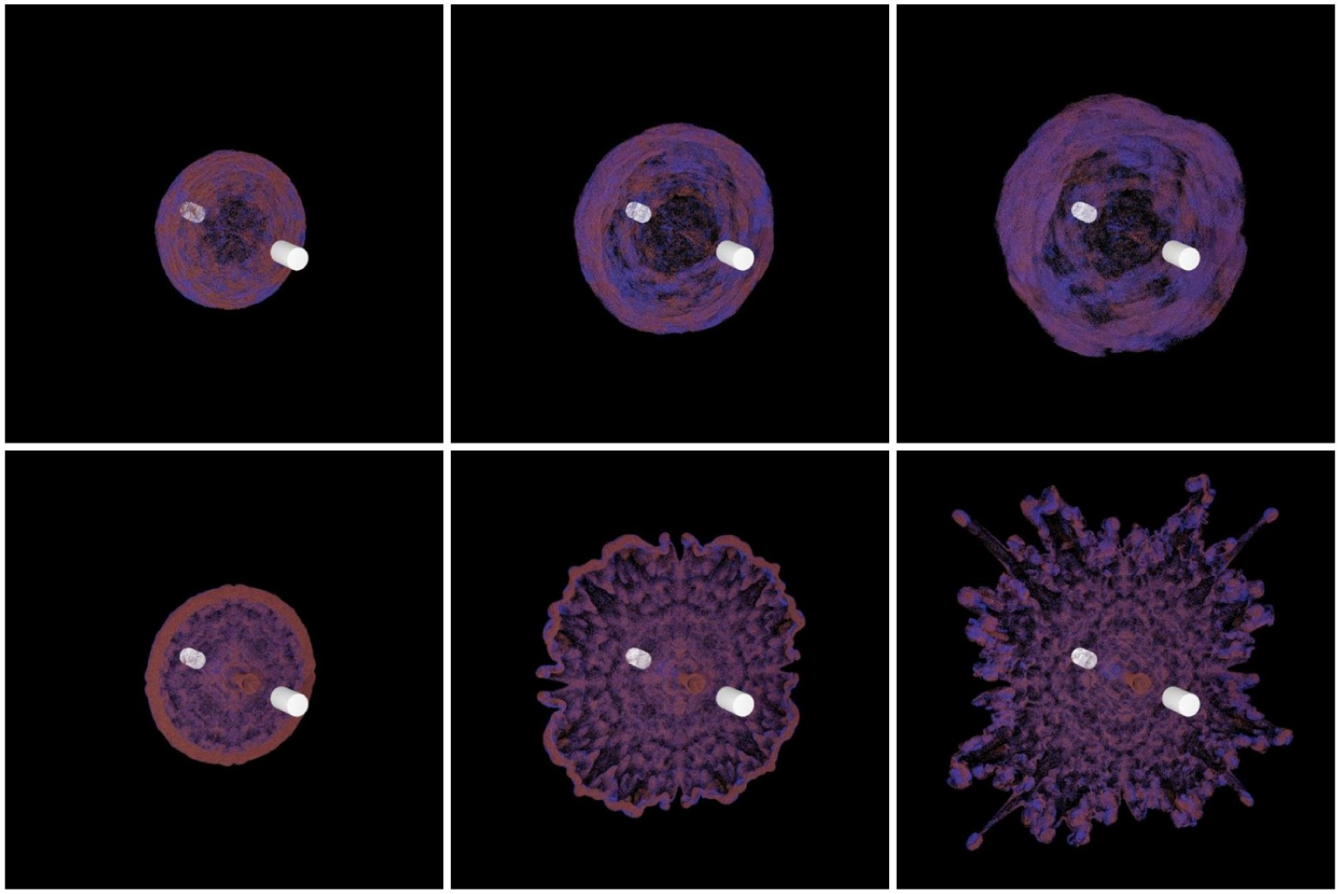
Colliding vortex rings using different methods. The first row: the MacCormack solver; the second row: our method.

Second, we use three different values of *δ* in the example of colliding vortex rings (see Fig 5). We set a uniform grid inside the ink and place the vortex particles in the center of every grid. Except for the normal, the vorticity distribution of the two inks is the same, which is calculated by applying Eq (18). We observe that the vortex re-connection phenomenon can be obtained after the collision of rings in all cases, which is consistent with the result reported in [37]. It proves that the numerical damping caused by *δ* does not change the physical nature of the simulation. When *δ* = 1, the vorticity is not suppressed. The vortices after re-connection show irregular motion and instability. In the case with smaller *δ*, the flow after the collision is more stable. This proves that our stretching approach can stabilize the simulation. In addition, we compare our approach to a MacCormack solver [14], as shown in Fig 6. The simulation with a MacCormack solver can not reproduce such phenomena.

### Nested grid results

#### Vortex ring

**Fig 7:**
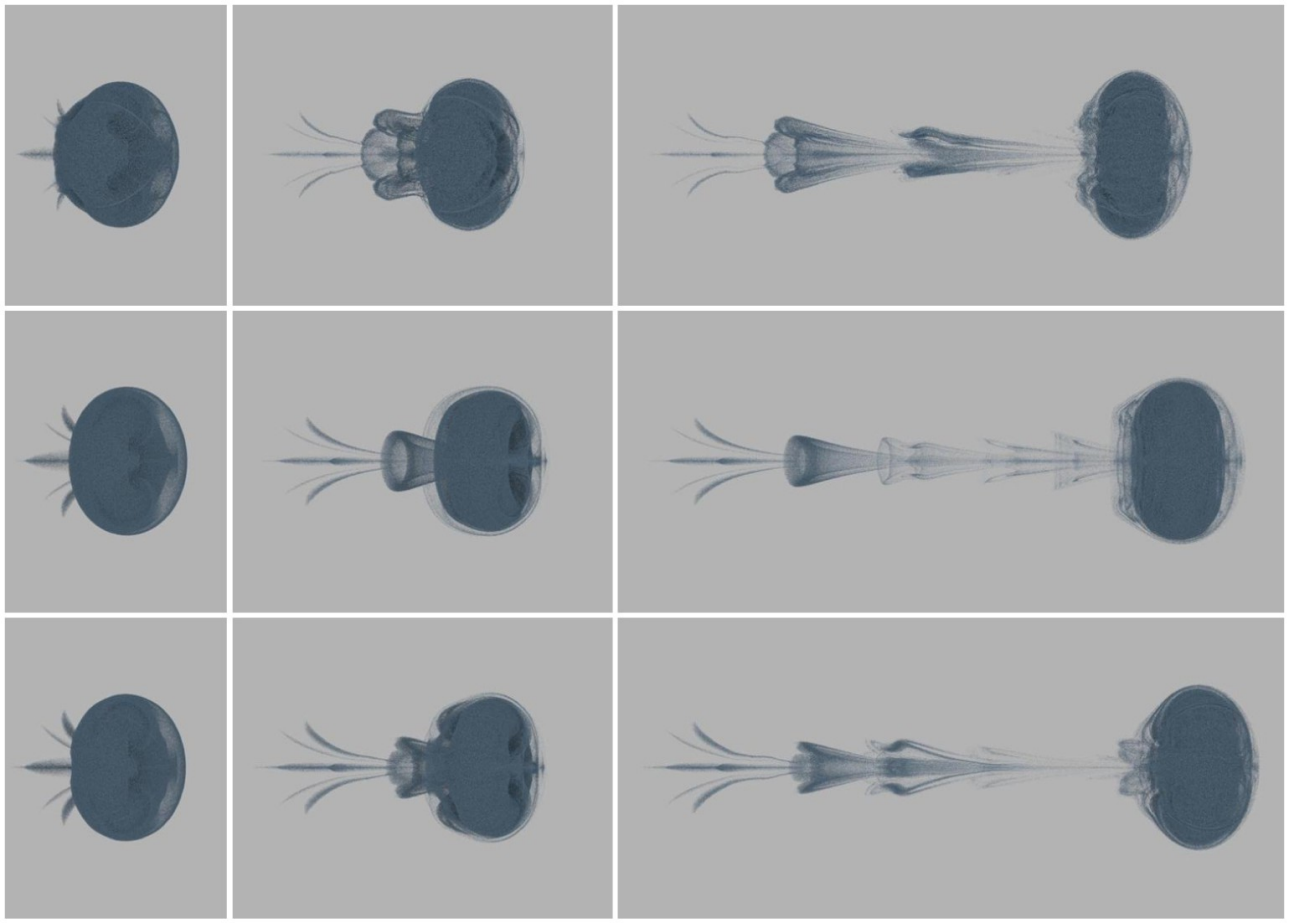
Vortex ring comparison of the octree grid method, the nested grid method and the direct summation method. The first row: the octree grid; the second row: the direct summation; the third row: our nested grid.

**Fig 8:**
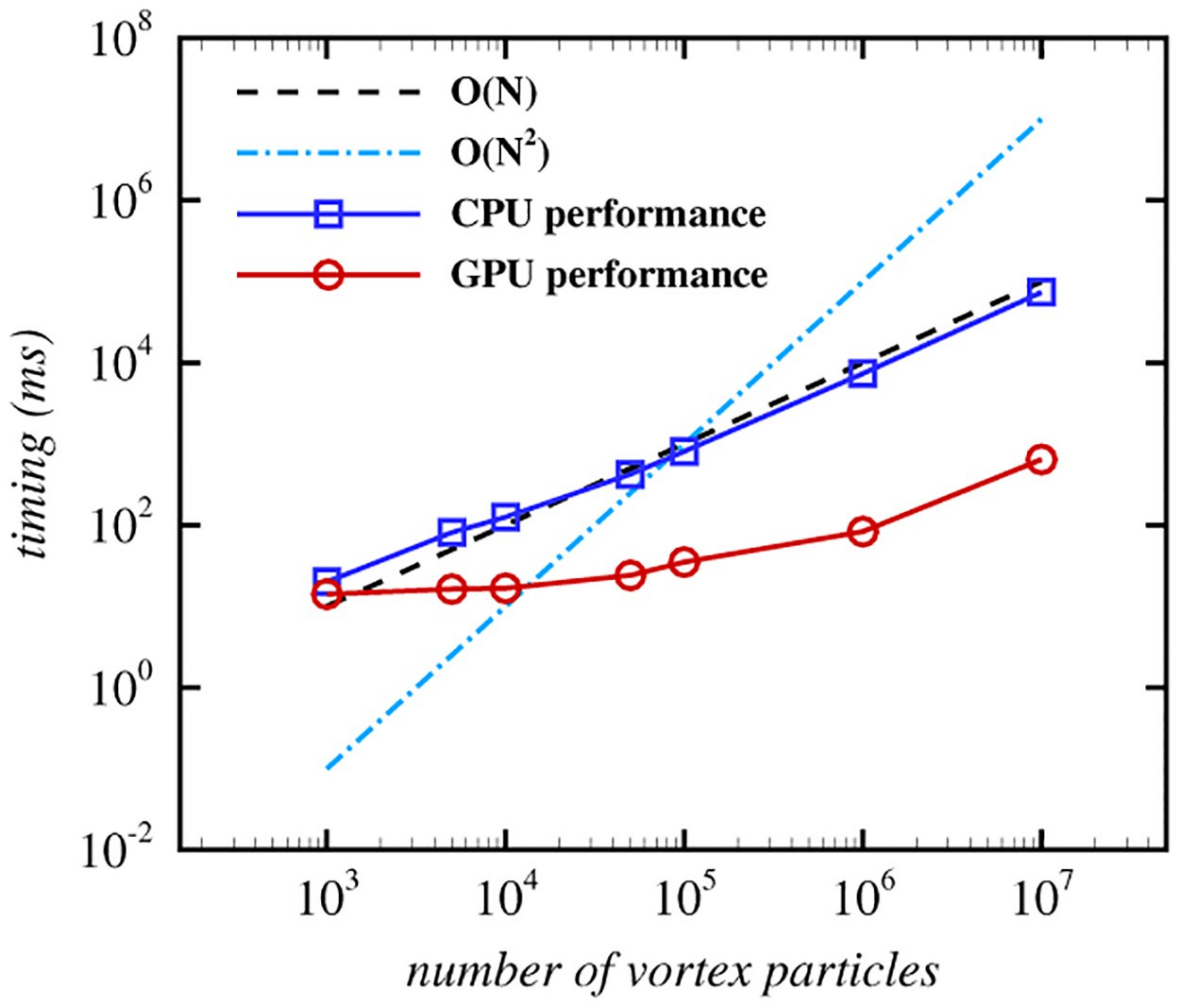
Performance of the nested grid method.

The simulation results of the octree grid [22], our nested grid and the direct summation are qualitatively compared by employing the vortex ring example. In the comparison, the initial conditions are set identically. The vorticity distribution of the vortex ring is approximated according to the empirical formula (18) [38]:

$$\omega (r)=\{\begin{array}{ll} \omega_{max}[1-\text{exp}(-\frac{K}{r^{*}}\text{exp}(\frac{1}{r^{*}-1})] & \text{if}\;r^{*}<1,\\ 0 & \text{else}, \end{array}$$ (18)

where r* = *r*/*R*, *R* is the radius of the cross-section of the vortex ring, *r* is the distance from the vortex particle to the vortex axis, and *K* = 1/2exp(2)log(2). The vorticity is concentrated in the core of the vortex ring, and the vorticity direction is tangent to the vortex axis and perpendicular to the normal of the vortex ring. The nested grid is a five-layer grid with the configuration of ${\left(K_{r}^{5}\right)}^{3}=64^{3}$. As depicted in Fig 7, our method’s simulation results are very similar to those of the direct summation method. Although some details are lost on the fluid surface and the vortex ring’s wake is slightly deformed, our method requires significantly less computation. And compared with the octree grid, our nested grid is visually closer to the original flow. Fig 8 shows the nested grid method’s computation time on the CPU and GPU with the different number of vortex particles. As the number of vortex particles increases, the computational time of our method grows linearly, proving that the time complexity is *O*(*K*_*v*_).

### Boundary treatment result

#### Kármán Vortex Street

**Fig 9:**
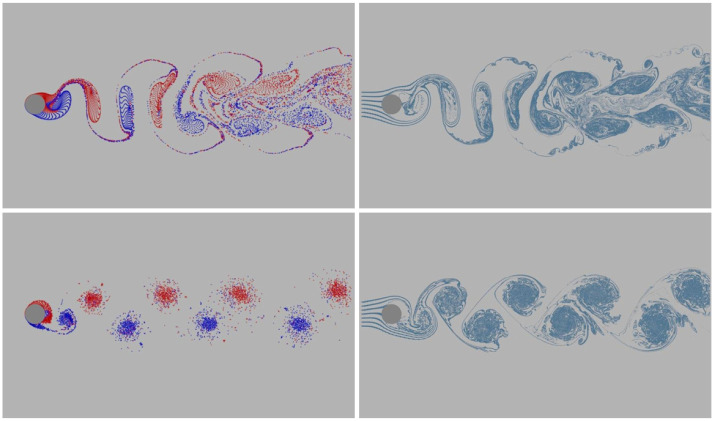
Comparison of Karman vortex street examples using different boundary treatment methods. The first row: the panel method; the second row: our boundary treatment method. Blue and red represent the vortex particles with negative and positive vorticity, respectively. Mineral blue represents the tracer particles.

**Fig 10:**
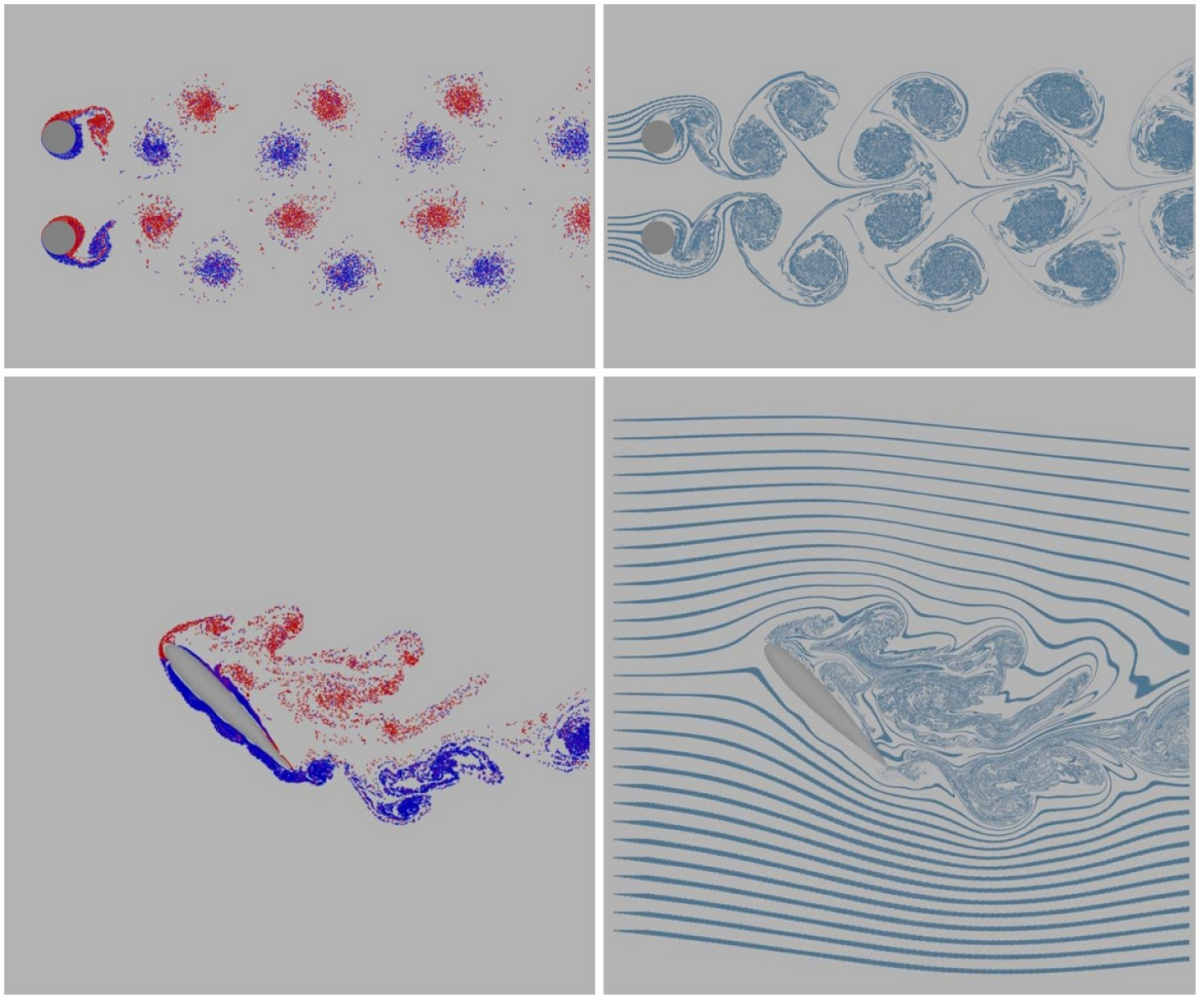
2D boundary treatment results. The first row presents Kármán Vortex Street behind two discs; the second row displays vortex shedding behind an airfoil. Blue and red represent the vortex particles with negative and positive vorticity, respectively. Mineral blue represents the tracer particles.

Fig 9 shows the simulation of classic fluid phenomena, Kármán Vortex Street. In this example, we compare our method with the panel method [31]. At the boundary of the disc, we sample 200 panels with the panel method and 200 boundary points with our boundary treatment method. As shown in Fig 9, some vortex particles that enter the obstacle’s interior are eliminated after advection. The generated vortex particles passing the obstacle produce a repeating pattern of swirling vortices, forming the Kármán vortex street phenomenon. Under the influence of viscosity, the vorticity strength of the vortex particles that are far away from the obstacle decreases slowly, and their velocities gradually become similar to the velocity of the background flow. However, the vortices in the panel method can not maintain for a long time, and finally merge together. This comparison showcase the effectiveness of our boundary treatment method. In addition, we simulate the vortex shedding behind two discs and an airfoil, as shown in Fig 10. We sample 200 boundary points around every disc and 400 boundary points around the airfoil. The airfoil’s angle is 45°. Compared with the disc, the flow passing around the airfoil presents a more irregular motion. The simulation shows that our method can handle the boundary of the obstacles with complex geometry.

**Fig 11:**
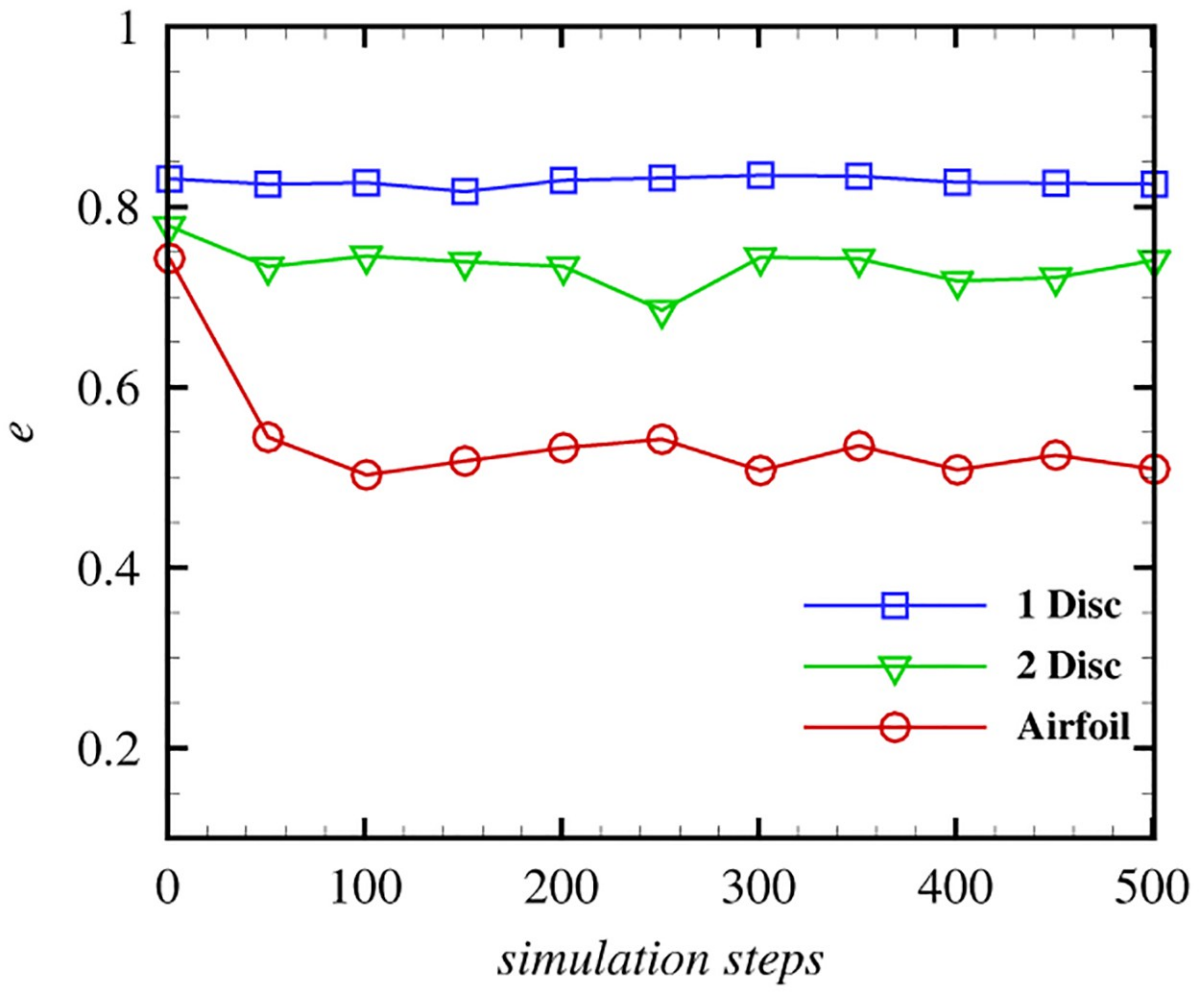
Comparison of the elimination ratio with different 2D obstacles.

In addition to the visual results, we provide the computation accuracy of the three cases presented in Fig 11. We define the ratio of velocity elimination as follows:

$$\begin{array}{c} e=\sum_{i=1}^{K_{g}}\frac{\left\|u_{f}^{i}+u_{\infty }^{i}-u_{b}^{i}\right\|-\left\|u_{f+}^{i}+u_{\infty }^{i}-u_{b}^{i}\right\|}{\left\|u_{f}^{i}+u_{\infty }^{i}-u_{b}^{i}\right\|}, \end{array}$$ (19)

where *K*_*g*_ is the number of boundary sampling points, *u*_*f*_ and *u*_*f*+_ are the velocity of fluid at the boundary sampling points before and after boundary treatment, respectively. *u*_∞_ is the velocity of the background flow, and *u*_*b*_ is the velocity of the solid at the boundary sampling points. In the Kármán Vortex Street example, the solid is static and the velocity of the background flow is constant. Therefore, *e* can be simplified as $e=\sum_{i=1}^{K_{g}}\frac{\left\|u_{f}^{i}\right\|-\left\|u_{f^{+}}^{i}\right\|}{\left\|u_{f}^{i}\right\|}$.

Fig 11 shows the ratio of the velocity elimination for the three cases. We can observe that our boundary treatment method can cancel out most of the slip and through velocities while dealing with regular obstacles. In the three cases, one disc achieves the highest accuracy, whose elimination ratio reaches approximately 83%. The accuracy of the airfoil is the lowest. We remark that our method is more suitable for dealing with the boundary of obstacles without sharp shapes.

#### Vortex shedding behind a static ball

**Fig 12:**
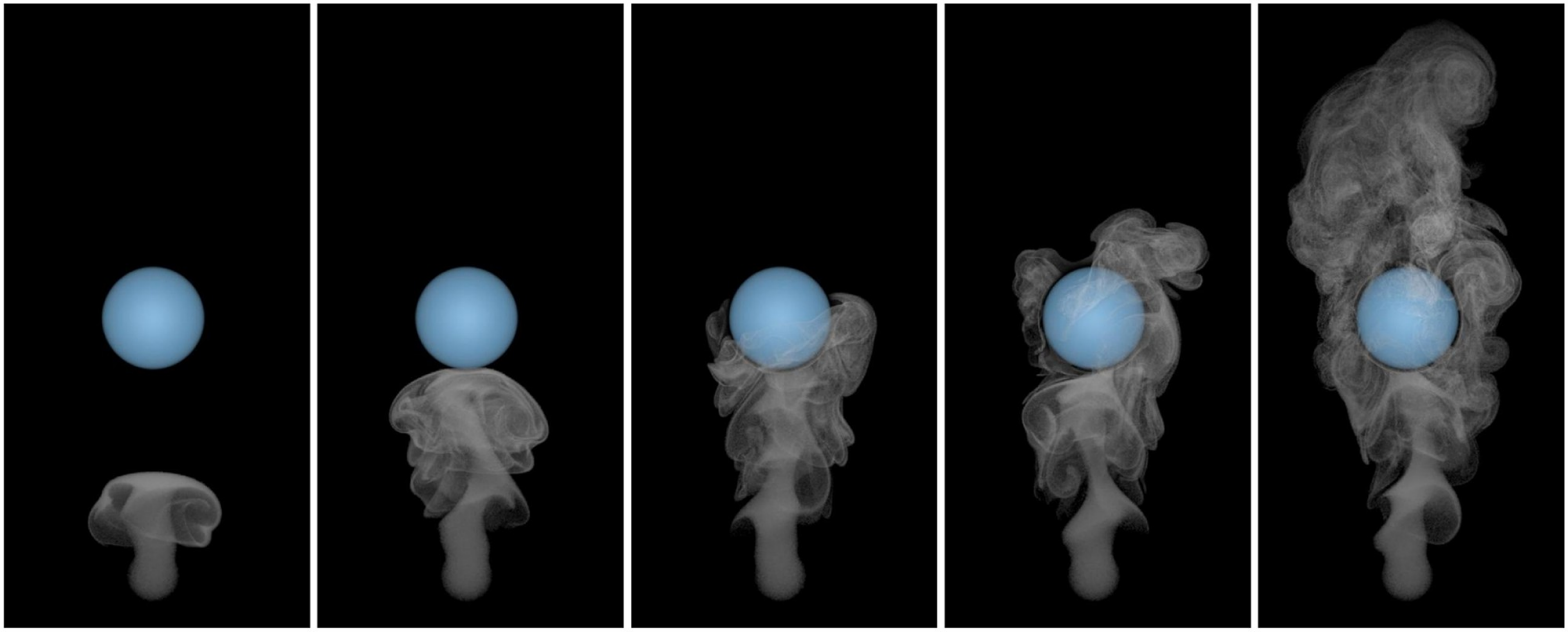
Smoke passing a static ball.

Fig 12 indicates the realistic wakes behind a static ball in the rising smoke example. In this experiment, we emit a vortex circle every 10 time steps, consisting of 200 vortex particles. The vortex circle carries the tracer particles upward. When the static ball is passed, the vortex particles entering the ball are destroyed, and the collision point with the obstacle is the boundary sampling point. The radius of the ball is 0.5 unit lengths.

**Fig 13:**
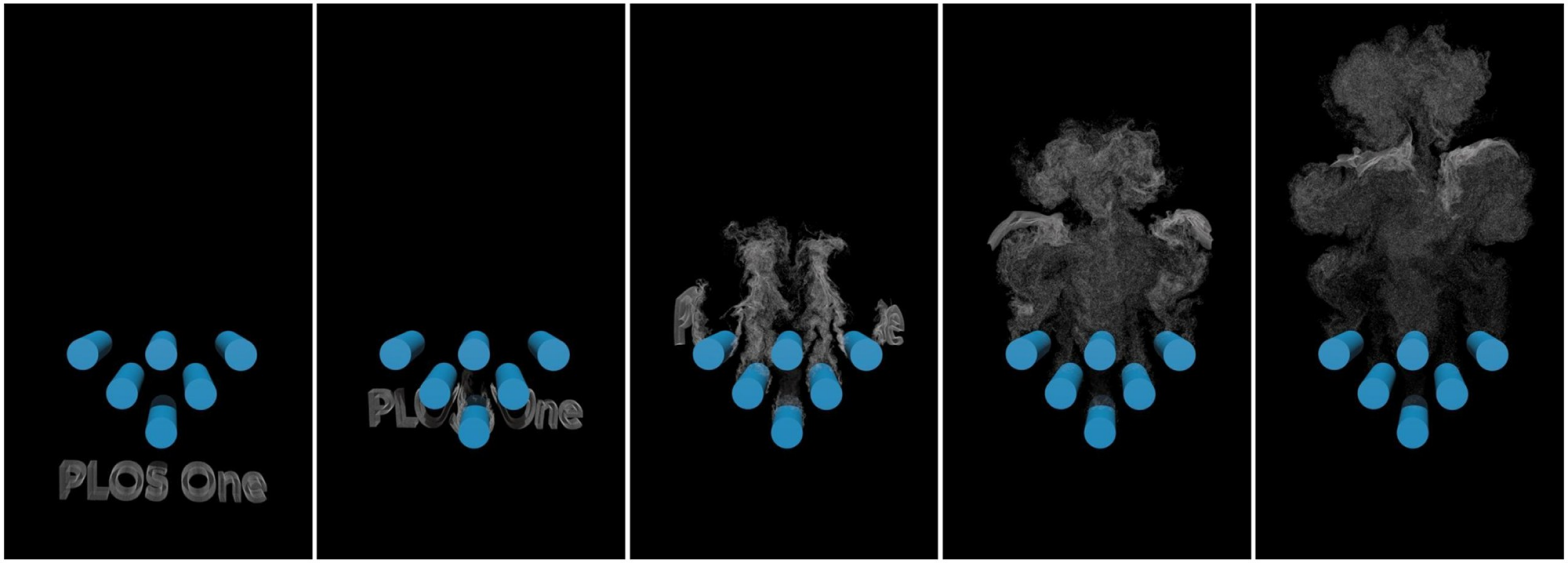
Smoke forming the words ‘PLOS One’ and passing through some obstacles.

Fig 13 demonstrates the realistic wakes behind the static cylinders. We construct the thin smoke to form the words ‘PLOS One’ and let it pass through the cylinders. The radius of the cylinder is 0.8 unit lengths. In this example, the boundary sampling point is computed in advance like the 2D Karman vortex street example. The vortex particles entering the cylinder are destroyed. The simulation results show that our method can produce dispersive thin smoke.

#### Vortex shedding behind moving objects

**Fig 14:**
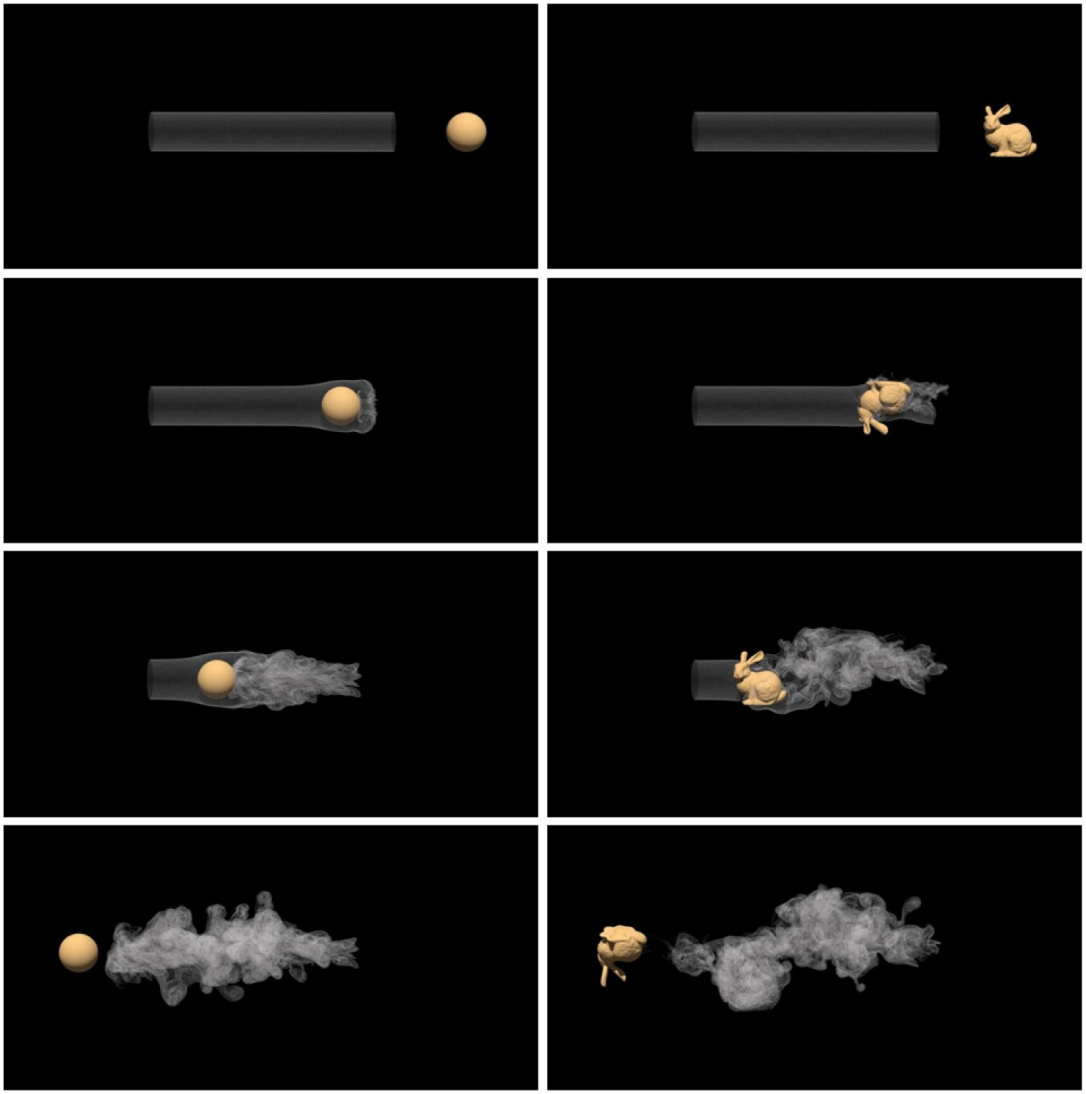
Moving object passing through smoke. The first column is a translation ball. The second column is a bunny with translation and rotation.

The first column in Fig 14 depicts the turbulent wake formed by the moving ball passing through the static smoke. Unlike in the 2D Karman vortex street’s sampling manner, we place massive vortex particles regularly in the static smoke and treat the collision points between the vortex particles and the moving ball as the boundary sampling points. The vortex particles entering the obstacle are destroyed. This means that the number of vortex particles is constant in the simulation. The ball’s radius is 0.5 unit lengths, and the ball moves at a speed of 2 unit-lengths/s.

The second column in Fig 14 presents the turbulent wake formed by the moving and rotating bunny passing through the static smoke. In this example, the boundary sampling manner is the same as the moving ball. We use the signed distance field to calculate the bunny’s normal and detect the collision between the vortex particles and the bunny. In addition to the translation speed, we introduce a rotation speed to the bunny to prove that our method can handle complex boundary conditions. Fig 14 shows that the bunny produces more details than the ball. The bunny’s size is equivalent to that of the ball, the translation speed is 2 unit-lengths/s, and the rotation speed is 2 unit radians/s.

### Performance

We parallelize most of our method’s steps using OpenMP and implement a GPU version of the nested grid method using CUDA, which is the most computationally expensive part of our method. Our examples are performed on a laptop with a 6-core 2.20 GHz CPU and a NVIDIA GeForce GTX 1060 graphics card. Table 1 lists the timing statistics of various smoke examples.

**Table 1:** Performance statistics of smoke simulations.

Figures	2D Scenes	Vortex No.	Time(ms)
Bottom in Fig 9	Kármán Vortex Street	20000	90
Top in Fig 10	Flow passing 2 discs	40000	200
Bottom in Fig 10	Flow passing a airfoil	40000	210
Figures	3D Scenes	Vortex No.	Time(ms)
Bottom in Fig 6	Colliding vortex rings	40000	70
Bottom in Fig 7	Vortex ring	64000	80
Fig 12	Smoke passing ball	80000	230
Fig 13	Smoke passing cylinders	120000	610
Right in Fig 14	Ball passing smoke	50000	175
Left in Fig 14	Bunny passing smoke	50000	225

## Conclusions

We presented a pure Lagrangian vortex method to produce rich details for smoke animation. Our method is efficient and suitable for parallelism on a GPU, thereby indicating its potential to be applied in real-time applications. This framework has two essential components. A nested grid is used to group the vortex particles and effectively reduce the computational cost of the BS law. This is validated by using the vortex ring example. Further, we proposed a novel vortex-generating method that supports dynamic boundary conditions for handling complex obstacles. We simulated a series of complex gas phenomena, such as the Kármán Vortex Street, colliding vortex rings, and interaction of smoke and obstacles.

Future work: The nested grid loses some accuracy when computing the velocity. We can improve this by dividing a direct summation area in the finest grid layer, while the other layers remain unchanged. Moreover, the number of generated vortex particles determines the linear equation’s size, making our method challenging in dealing with the interaction between smoke and large-scale obstacles. Reducing the linear equation’s size or splitting the equation into multiple sub-equations could be beneficial. Finally, the two-way fluid rigid body interaction is a prominent research direction for future work.

## Supporting information

S1 Video (MP4)

S1 Fig Overview of our vortex method. (TIF)
