# Supplementary figures and images for: Research on smoke simulation with vortex shedding
Source: PLoS One. 2022 Jun 16;17(6):e0269114. doi: 10.1371/journal.pone.0269114 (PMC9202895; doi:10.1371/journal.pone.0269114)

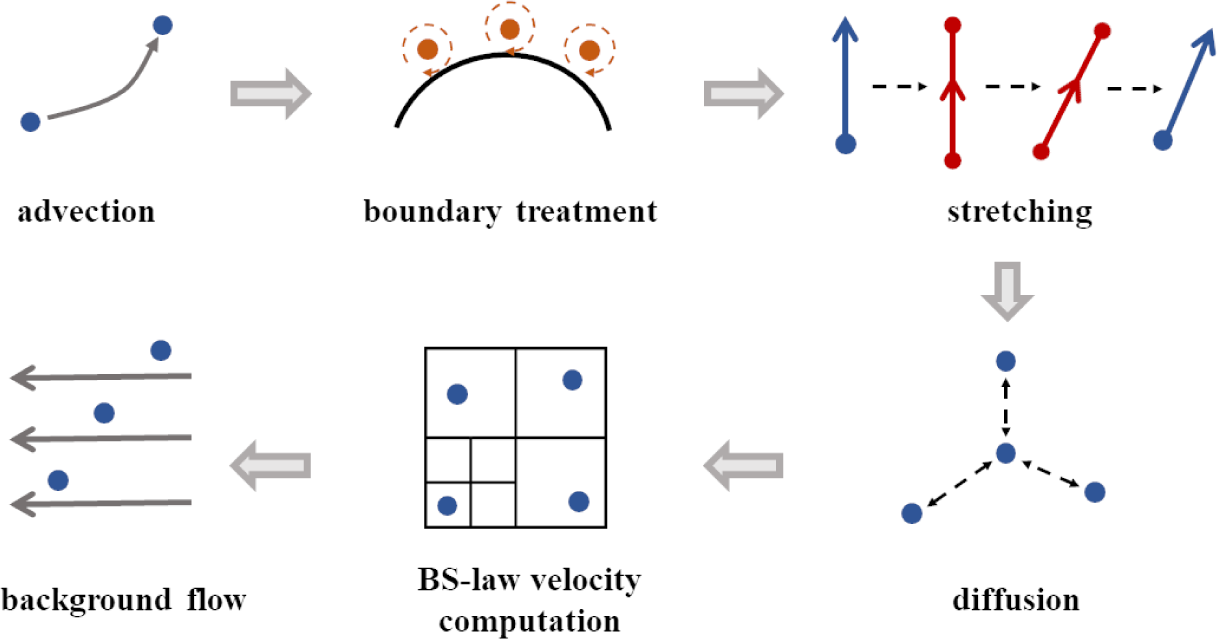

Supplement: S1 Fig — (TIF) [file pone.0269114.s002.tif]
